# Effect of screw access hole or vent hole opening strategies on the adhesive filling rate of oral implant cement-retained posterior crowns

**DOI:** 10.1371/journal.pone.0323092

**Published:** 2025-05-15

**Authors:** Long Li, Xiaodong Sun, Huangjun Zhou, Min Liu, Cai Wen

**Affiliations:** 1 Department of Prosthodontics, The Affiliated Stomatology Hospital, Southwest Medical University, Luzhou, China; 2 Department of Oral Implantology, The Affiliated Stomatology Hospital, Southwest Medical University, Luzhou, China; 3 Luzhou Key Laboratory of Oral & Maxillofacial Reconstruction and Regeneration, Southwest Medical University, Luzhou, China; 4 Institute of Stomatology, Southwest Medical University, Luzhou, Sichuan, China; 5 Department of Dentistry, Chengdu Pidu District People’s Hospital, Chengdu, China; International Medical University, MALAYSIA

## Abstract

**Purpose:**

This study investigated the adhesive filling rate (AFR) of implant prostheses on the abutment shoulder and axial surfaces under various hole opening strategies,and examined the correlation between hole openings and permanent adhesive retention strength.

**Methods:**

Implant crowns were divided into five groups: No hole (NH); Occlusal regular hole (ORH: 2.5 mm); Occlusal mini-hole (OMH: 1 mm); Lateral upper mini-hole (LUMH: 1 mm); and Lateral down mini-hole (LDMH: 1 mm). In the AFR experiment, abutments and prostheses were connected with two-color silicone rubber; the coverage of rubber at the inner surface of the prostheses was photographed. Images were analyzed by ImageJ software. In the adhesive retention strength experiment, prostheses and abutments were bonded using permanent resin cement; retention strength was measured using a universal testing machine. Data were analyzed using one-way analysis of variance (ANOVA) or Welch’s ANOVA, followed by Tukey’s honestly significant difference test.

**Results:**

Abutment shoulder AFRs were OMH (98.70 ± 0.42%), LDMH (98.40 ± 1.30%), LUMH (97.92 ± 1.33%), NH (93.99 ± 5.45%), and ORH (86.11 ± 4.90%). One-way ANOVA revealed significant difference among groups (p < 0.001). Axial AFRs were LUMH (99.2 ± 0.47%), ORH (98.3 ± 0.8%), OMH (98.1 ± 0.5%), LDMH (97.9 ± 1.06%), and NH (96.4 ± 4.5%),Welch’s ANOVA indicated no significant difference between groups (p = 0.054). In the retention strength experiment, OMH had the highest retention force (369.58 ± 27.27 N), whereas ORH had the lowest (272.81 ± 41.43 N), showing significant differences (p = 0.002).

**Conclusions:**

Implant screw access hole or vent hole opening strategies affected AFR of implant cement-retained posterior crowns. Larger holes or no openings decreased AFR at the abutment shoulder, whereas axial AFR was less affected. Hole openings variations on implant cement-retained posterior crowns might also influence their retention strength.

## Introduction

Dental implants are a reliable method for replacing missing teeth and represent an essential treatment option [1–4]. Implant prostheses can be primarily categorized into cement-retained or screw-retained based on superstructure retention [5–8]. Cement-retained prostheses involve fixing the upper implant restoration to the abutment using bonding materials such as glass ionomer cement or resin cement. Screw-retained prostheses involve securing the upper implant restoration to the abutment with a fixed screw [9–12].

Cement-retained prostheses offer advantages such as simple fabrication, ease of operation, and passive seating [13,14]. However, they may dislodge if the bond strength is insufficient [15]. Residual adhesive at the abutment shoulder has also been identified as a risk factor for peri-implant diseases [16–18]. Research has shown that using occlusal overflow holes for implant prostheses can reduce cement spillage at the abutment margins [19]. In addition, transferring the vent hole of the implant crown to the lateral side of crown can also reduce the adhesive overflow, while avoiding esthetic defects caused by opening the occlusal surface [20]. However, hole-opening strategies are not ideal, as these holes can compromise the integrity and esthetics of the occlusal surfaces. The increase in hole diameter has been reported to be associated with a decreased retention force when bonded with temporary adhesives[19,20]. However, there is no conclusive evidence regarding retention changes with different hole diameters and positions when using permanent adhesives.

The effect of adjusting the implant crown opening on retention may involve changes in the bonding area and intraprosthetic adhesive filling rate (AFR). AFR was defined as the ratio of the area covered by the adhesive inside the crown after bonding to the total area inside it. The influence of implant prosthesis hole openings on the AFR inside the crown has not been systematically investigated. To address this, our team proposed optimization schemes for hole-opening strategies, such as appropriately reducing hole diameters or relocating them to the lateral sides of the prostheses. Therefore, this study aimed to analyze the AFR of the abutment shoulder and axial inner surfaces of implant prostheses with different opening hole diameters and positions, and determine its effect on bonding strength.

## Methods

### Preparation of abutment base

Titanium made implant abutment (Dentium, Korea) was connected with an analog and tightened at 35 Ncm torque. The abutment analog specimen was then inserted into a mold. Epoxy resin (Ruijuan, China) was poured into the mold and left to completely solidify.

### Design and preparation of implant restoration

**Fig 1:**
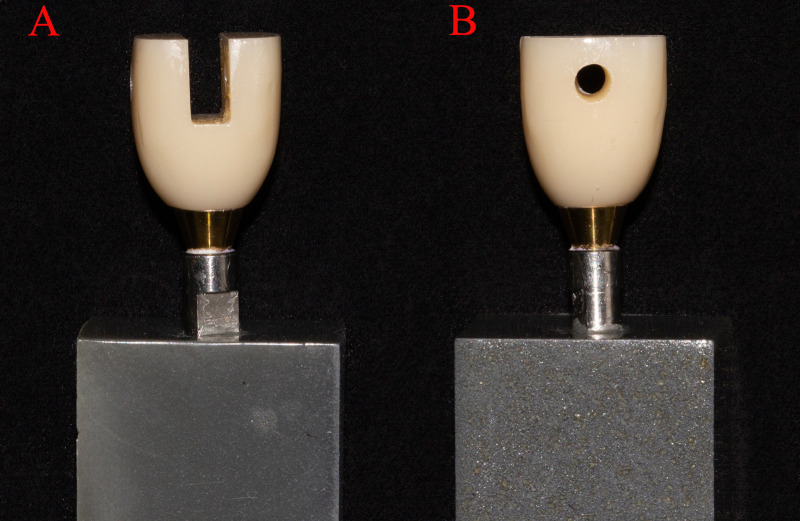
(A) Side image of epoxy resin-made abutment-analog base and prosthesis; (B) Front image of epoxy resin-made abutment-analog base and prosthesis.

The implant prostheses were designed using computer-aided design systems (exocad, Germany). The occlusal surface of the restoration was designed to be flat to facilitate pressure loading. Two traction holes perpendicular to the long axis of the restoration were included to facilitate tensile traction (Fig 1).

**Fig 2:**
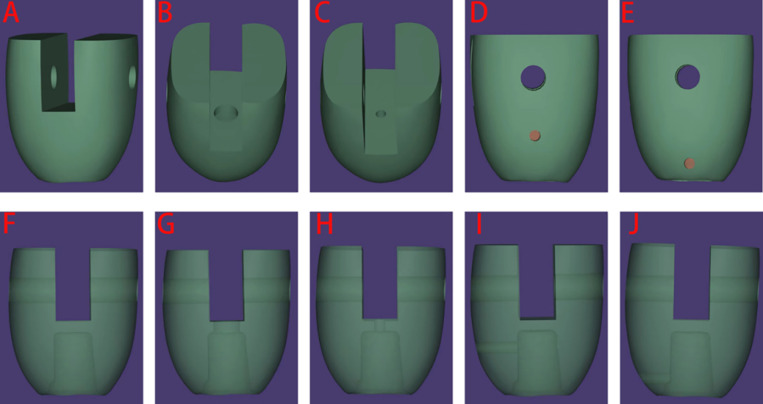
Front and side images of the computer-aided design of the prostheses, including an interior perspective showing the location and size of the holes. (A and F) Images of no hole (NH); (B and G) Images of occlusal regular hole (ORH); (C and H) Images of occlusal mini-hole (OMH); (D and I) Images of lateral upper mini-hole (LUMH); and (E and J) Images of lateral down mini-hole (LDMH: 1 mm).

**Fig 3:**
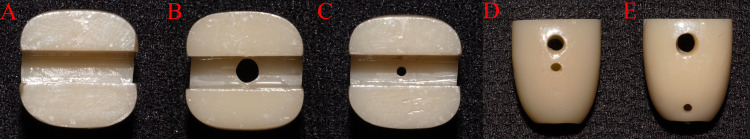
Photograph of the implant prostheses with different hole opening strategies. (A) NH occlusal view; (B) ORH occlusal view; (C) OMH occlusal view; (D) LUMH lateral view; and (E) LDMH lateral view.

Based on the presence, size, and location of the holes, the prostheses were categorized into five groups: A, No hole (NH); B, Occlusal regular hole (ORH: 2.5 mm); C, Occlusal mini-hole (OMH: 1 mm); D, Lateral upper mini-hole (LUMH: 1 mm); and E, Lateral down mini-hole (LDMH: 1 mm).The cement space between the restoration inner fitting surface and abutment was set to 20 μm. The same dental laboratory technician (Taimei, China) fabricated 25 cement-retained zirconia prostheses using the design below,and each group comprised five prostheses (Figs 2 and 3).The inner wall of the finished implant crown is routinely sandblasted.The sample size was set to 5 specimens per group (totaling 25 specimens) due to costs associated with fabricating prosthetic crowns and abutments. This constraint was carefully weighed against the need for statistical robustness, and the selected sample size aligns with precedent studies evaluating comparable biomechanical properties in dental prostheses [21,22].

### Adhesive filling rate test

**Fig 4:**
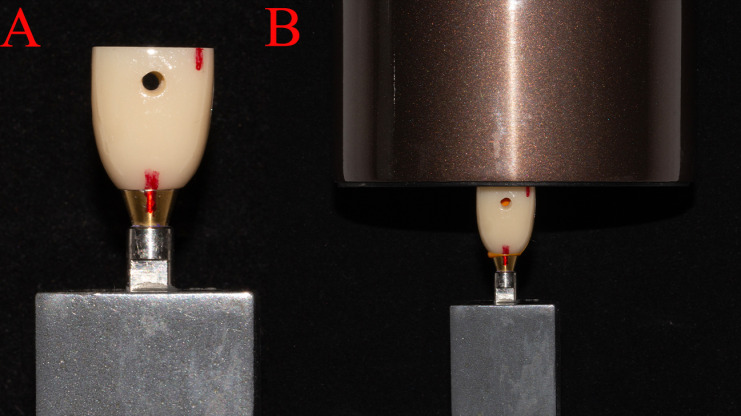
(A) Auxiliary marking points to ensure complete and accurate seating of the prosthesis. Red lines marked on the surface of the prosthesis and abutment are fully connected, indicating that the prosthesis is passively seated. (B) Standardized weight pressuring after prosthesis’s passive seating.

Prostheses and abutments were assembled, and full and complete seating was ensured. Seating points at the interface between the four axial surfaces of the prosthesis and abutment were marked. A double-color silicone rubber method was employed to simulate the bonding process. Yellow high fluidity silicone rubber was deemed to represent the adhesive, whereas blue low fluidity silicone rubber indicated unfilled areas. An equal amount (40 mg) of the mixed yellow silicone rubber (DMG, Germany) was evenly applied to the restoration’s inner surface. After fully seating of the prosthesis on the abutment according to the outer surface markings, a 2 kg weight was placed on it (Fig 4).

**Fig 5:**
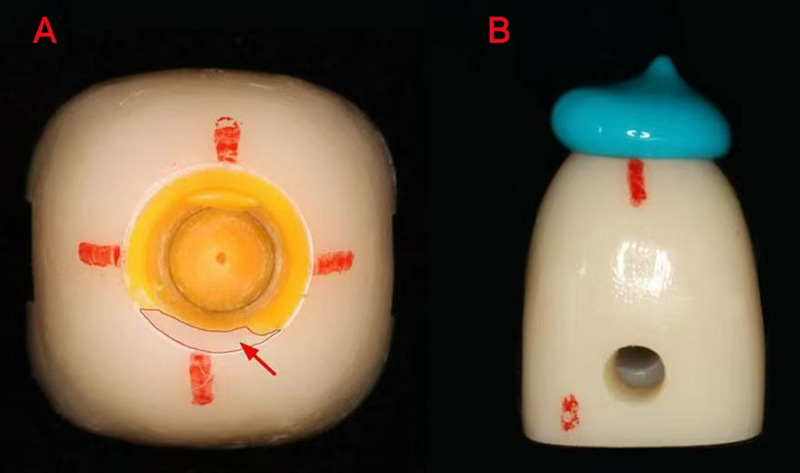
(A) Yellow high fluidity silicone rubber is in the inner surface of the prosthesis, and red arrow shows the unfilled part. (B) Blue low fluidity silicone rubber is injected into the prosthesis.

After the yellow silicone rubber completely solidified, the abutment and prosthesis were slowly separated by hand, and gentle motions were applied to ensure the silicone material within the crown was not dislodged. Blue low fluidity silicone rubber (DMG, Germany) was then injected into the inner surface to fill remaining unfilled areas (Fig 5). Subsequently, the prosthesis was connected with abutment following the same protocol as previously described. The marking points were carefully aligned to ensure precise connection, followed by re-application of weight for standard compression.After complete solidification, the silicone rubber model was removed from the prosthesis with the aid of a dental probe and tweezers.The probe was gently used to break the marginal seal of the silicone rubber, and then the tweezers were employed to extract it completely.

**Fig 6:**
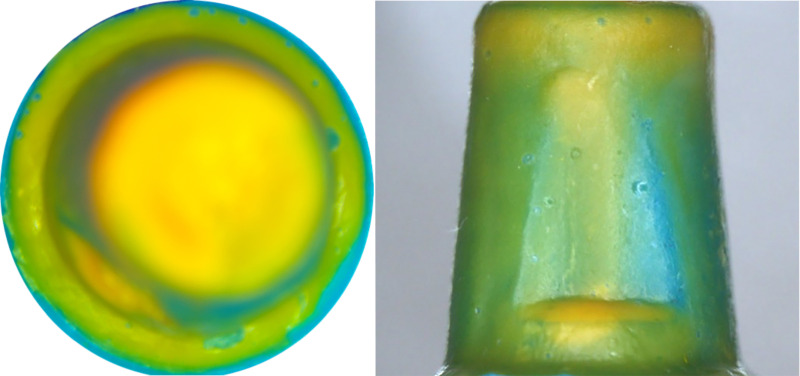
Coverage of silicone rubber on the axial and abutment shoulder surfaces (Yellow: high fluidity rubber; Blue: low fluidity rubber).

**Fig 7:**
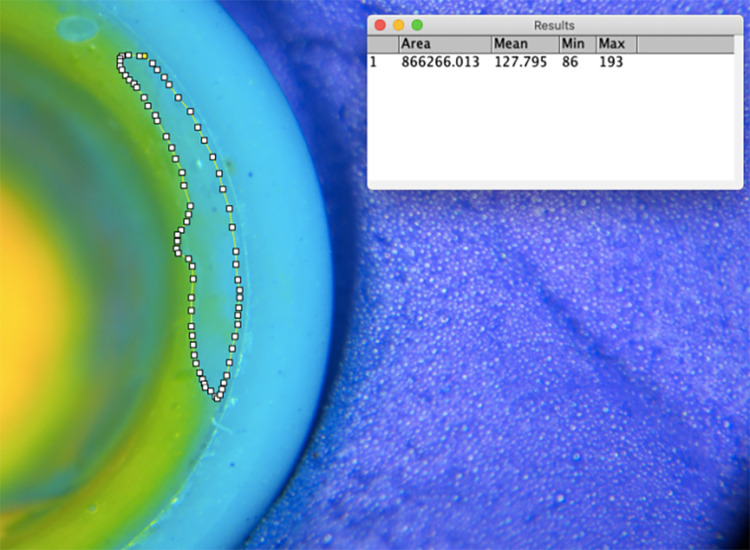
Abutment shoulder adhesive filling rate (AFR) of silicone rubber calculated by ImageJ.

A stereomicroscope (Shunyu Optics, China) with 15 × magnification was used to observe and photograph the abutment shoulder and axial surface coverage of the silicone rubber film (Fig 6). The photographs were imported into ImageJ (National Institute of Health, USA) to calculate the abutment shoulder and axial surface adhesive filling rates (Fig 7).

Abutment shoulder AFR (%) = silicone rubber covered area on abutment shoulder/abutment shoulder total area×100%

Axial surface AFR (%) =silicone rubber covered area on axial surface/axial surface total area×100%

### Retention strength test

The permanent adhesive retention strength of the prosthesis was assessed by measuring the tensile force required to separate the prosthesis from the abutment. After AFR tests, the prostheses and abutments were cleaned with alcohol cotton pellets, and then air dried for 5 sseconds. Permanent resin cement U200 (3M ESPE, USA) was used to bond the crown to the abutment. After fully seated the crown, the 2 kg weight was also applied to the occlusal surface of each crown for 30 seconds. the overflow resin cement was cleaned by a dental probe,and the cement was light-cured for 20 seconds on the buccal, occlusal, and lingual surfaces.

**Fig 8:**
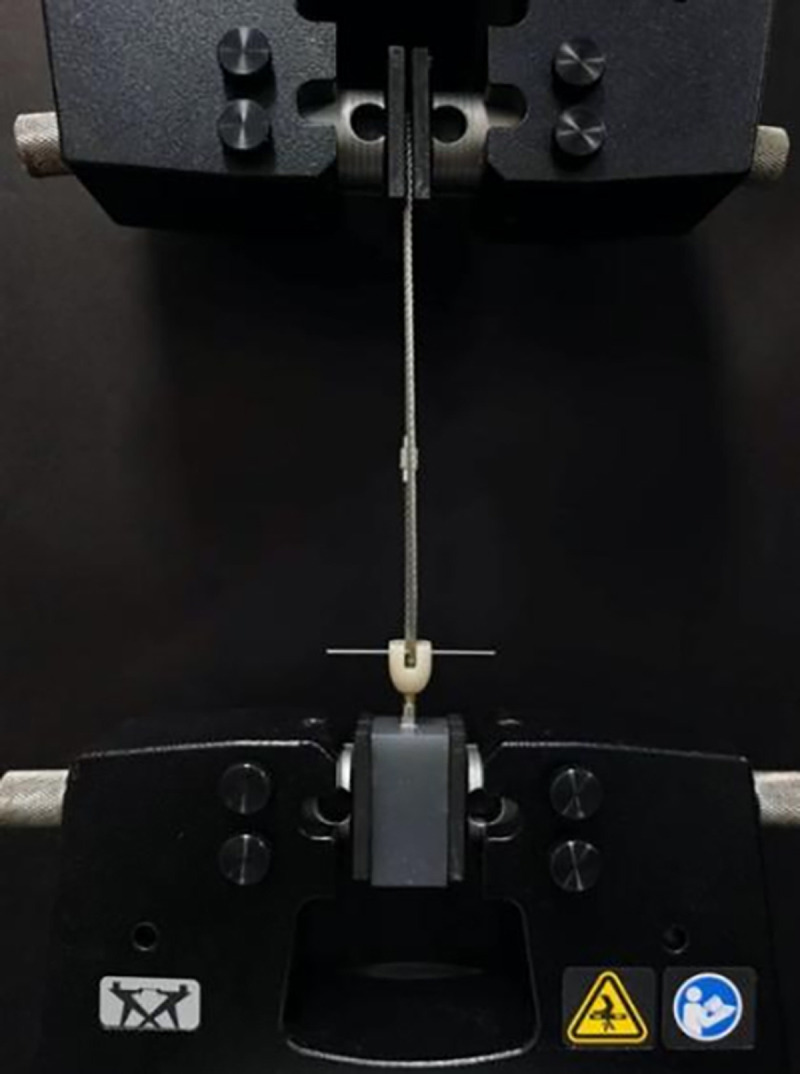
Retention strength testing of the cemented crown by universal testing machine.

Bonded tensile forces were measured using an electronic universal testing machine (Instron, USA) (Fig 8). Tensile force was applied at a rate of 1 mm/min parallel to the long axis of the specimen until complete separation. The maximum tension value from the time-tension curve was recorded as the bond retention strength(N).Tensile experiments were performed separately for each group of prostheses, with abutments cleaned and dried after each experiment.

### Statistical analysis

SPSS 23.0 statistical software (IBM, USA) was used to analyze the experimental data. Means and standard deviations were calculated. The Shapiro-Wilk test was employed to evaluate the normal distribution of the data, while Levene’s test was utilized to examine the homogeneity of variances. One-way analysis of variance (ANOVA) was used to analyze differences between groups if the data variance was homogeneous; otherwise, Welch’s ANOVA was applied. Tukey’s honestly significant difference (HSD) test for multiple comparisons was used to determine between-group differences. Statistical significance was set at p < 0.05.

Since this article contains no studies involving human participants or animals, ethics approval was not required

## Results

### AFR at abutment shoulder

**Fig 9:**
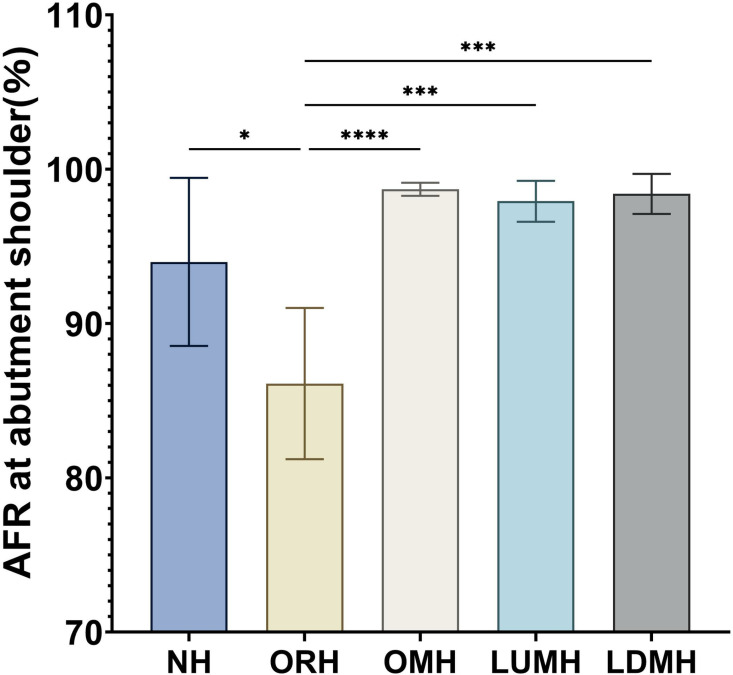
AFR at abutment shoulder in each group calculated by ImageJ software. *, P < 0.05; **;P < 0.01;***, P < 0.001;****, P < 0.0001.

The abutment shoulder exhibited the highest AFR in group OMH (98.70 ± 0.42%), followed by groups LDMH (98.40 ± 1.30%), LUMH (97.92 ± 1.33%), NH (93.99 ± 5.45%), and ORH (86.11 ± 4.90%) (Table 1 and Fig 9). Levene’s test indicated that the variances were homogeneous (F = 2.401, P = 0.084). One-way ANOVA revealed a significant difference in AFR among the groups (p < 0.001). The AFR in the ORH group was significantly lower compared with the other groups. Multiple intergroup comparisons of Tukey’s HSD test results showed that the AFR of the ORH group differed significantly from that of the other groups.

**Table 1:** Results of adhesive filling rate (AFR) at abutment shoulder filling rate in each group.

					95% Confidence Interval for Mean					
Group	Sample size	Mean	Std. Dev	Std. Error	Lower Bound	Upper Bound	Min	Max	F	P
NH	5	93.99	5.45	2.44	80.02	87.22	84.99	98.43	12.407	< 0.001^*^
ORH	5	86.11	4.90	2.19	80.02	92.20	77.66	90.22		
OMH	5	98.70	0.42	0.19	98.19	99.22	98.08	99.10		
LUMH	5	97.92	1.33	0.60	96.27	99.58	96.33	99.57		
LDMH	5	98.40	1.30	0.58	96.79	100.02	96.20	99.56		
Total	25	95.02	5.77	1.15	92.64	97.14	77.66	99.57		

*indicates significant difference.

### AFR of axial surface

The AFR of the axial surface in each group was: NH (96.46 ± 4.45%), ORH (98.33 ± 0.83%), OMH (98.17 ± 0.52%), LUMH (99.25 ± 0.47%), and LDMH (97.94 ± 1.06%) (Table 2 and Fig 10). Levene’s test indicated unequal variances (F = 4.502, P = 0.009) and Welch’s test revealed no statistically significant differences in axial AFR among groups (F = 3.434, p = 0.054). These results revealed that the diameter and position of the openings had a limited impact on the axial AFR of implant prostheses.

**Table 2:** Results of AFR at axial surfaces of implant prostheses in each group.

					95% Confidence Interval for Mean					
Group	Sample size	Mean	Std. Dev	Std. Error	Lower Bound	Upper Bound	Min	Max	F	P
NH	5	96.46	4.45	1.99	90.94	101.99	88.55	98.92	3.434#	0.054
ORH	5	98.33	0.83	0.37	97.30	99.37	97.60	99.61		
OMH	5	98.17	0.52	0.23	97.52	98.82	97.62	99.03		
LUMH	5	99.25	0.47	0.21	98.67	99.83	98.51	99.70		
LDMH	5	97.94	1.06	0.47	96.63	99.25	96.83	99.45		
Total	25	98.03	2.13	0.43	97.15	98.91	88.55	99.70		

# indicates Welch’s ANOVA

**Fig 10:**
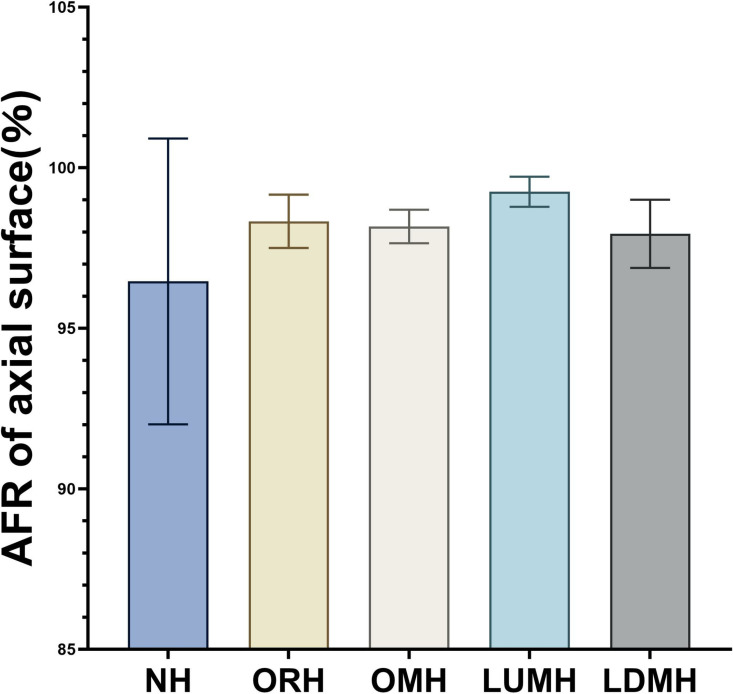
AFR of axial surface for each group calculated by ImageJ software.

### Retention strength with permanent adhesive

**Fig 11:**
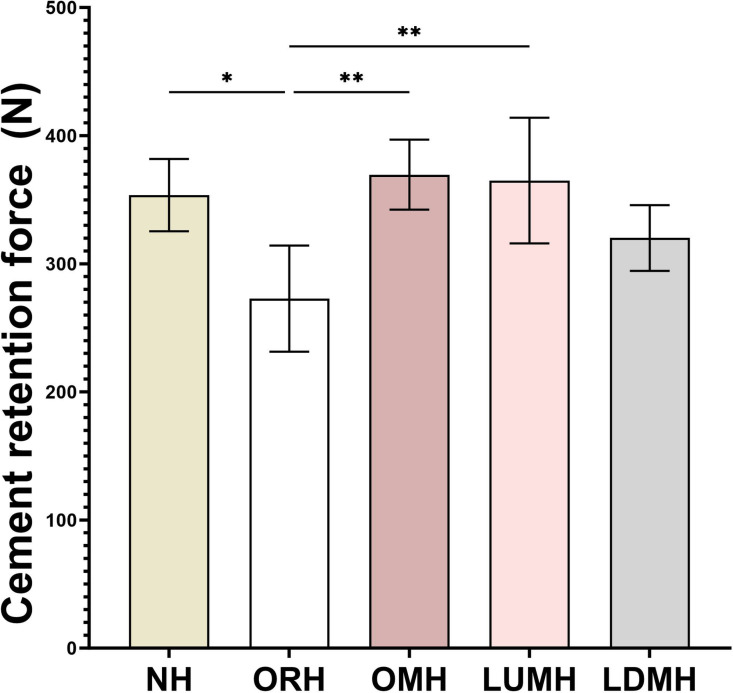
Cement retention force values(N) for each group in the cement retention strength test. *, P < 0.05; **, P < 0.01.

The highest retention force was observed in group OMH (369.58 ± 27.27 N), whereas the lowest was recorded in group ORH (272.81 ± 41.43 N) (Table 3 and Fig 11). Levene’s test indicated that the variances were equal (F = 0.538, P = 0.709), and one-way ANOVA showed a significant difference in retention strength between the experimental groups (p = 0.002). Significant differences were found between the ORH group and the other groups, except for LDMH, by Tukey HSD test. No statistically significant differences were found between the NH, OMH, LUMH, and LDMH groups.

**Table 3:** Results of cement retention with permanent adhesive in each group.

					95% Confidence Interval for Mean					
Group	Sample size	Mean	Std. Dev	Std. Error	Upper Bound	Lower Bound	Min	Max	F	P
NH	5	353.61	28.21	12.62	318.57	388.64	322.97	383.43	6.450	0.002^*^
ORH	5	272.81	41.44	18.53	221.36	324.27	222.91	326.72		
OMH	5	369.58	27.27	12.20	335.71	403.45	341.42	402.63		
LUMH	5	364.93	48.99	21.91	304.10	425.76	325.52	448.75		
LDMH	5	320.10	25.70	11.49	288.19	352.02	285.10	345.23		
Total	25	336.21	49.11	9.82	315.94	356.48	222.91	448.75		

*indicates significant difference.

## Discussion

Adhesive residues in cement-retained implant prostheses could lead to complications [23], such as inflammation of hard and soft tissues [24–27]. To minimize adhesive residue at the abutment shoulder, drilling overflow holes on the occlusal surface of the implant restoration is common practice [28,29]. Typically, these overflow holes are positioned at the center of the abutment, corresponding to the screw access hole.

However, in the anterior region, due to the incongruity between the axial orientation of the bone and implant body, screw access holes may be located incisally or labially, which can significantly affect the esthetics of the restorations. Previous studies have shown that patients have negative esthetic attitudes toward implant crowns with occlusal holes [30]. Furthermore, opening hole on the crown with larger-diameter may lead to repeated shedding of restorative material [19,31], increasing patient revisits and potentially weakening the structural integrity of the restoration [32,33]. The strategy of opening hole for the implant prosthesis significantly affects the amount of adhesive spillage, no matter its position,whereas the diameter of the holes does not [20].

The specific effect of opening holes on the internal adhesive filling status of prostheses remains inadequately understood. The importance of the AFR inside the restoration as a basis for ensuring retention should not be overlooked. A poor filling ratio causes a discontinuity in the adhesive layer between the restoration and abutment, reducing the bonding area, weakening retention strength [34], and increasing the risk of dislodgment during mastication. Specifically, poor filling at the abutment shoulder can lead to micro-leakage between the implant restoration and the abutment edge. The micro-leakage allows bacteria to accumulate and produce acid [35,36], accelerating resin degradation and resulting in inflammation of the surrounding hard and soft tissues [36–38], potentially leading to implant failure. Moreover, long-term chewing forces can create micro-movements at the interface, weakening the adhesive bond and leading to dislodgement of implant prosthesis [13], or, in severe cases, fracture of the abutment screw [39].

The current research methods for assessing AFR vary. Some studies have used computational fluid dynamics. Sun et al. [28] utilized computational fluid dynamics in conjunction with in vitro mechanical tensile experiments to investigate the effects of different cement retention forms on adhesive flow and restoration retention. Their results showed that either an occlusal surface hole or a lingual side hole could effectively reduce adhesive spillage at the margins. However, regarding the internal filling rate of the adhesive, the cement in the no holes group was severely underfilled. The discrepancy in the results may be attributed to variations in experimental methodologies.Computational fluid dynamics analysis is a computer simulation which can be significantly influenced by modelling methods and may not accurately reflect clinical reality. Therefore, this study used a modified two-color silicone rubber method to determine the AFR in a practical setting, aiming to better simulate the clinical bonding process.The silicone rubber was applied to the inner surface of the restoration, thereby simulating the actual bonding process and producing results that more closely align with clinical practice.

The results for the abutment shoulder filling rate showed that, except for the larger 2.5-mm hole, the AFR in the opening hole group was significantly higher than that in the NH group, indicating that opening holes were beneficial for achieving a tight adhesive fill. This may be due to the fact that, without an air overflow hole, air trapped inside the crown cannot be discharged during bonding [28]. This results in increased air pressure, which reduces the contact area between the abutment and prosthesis, leading to a lower AFR.

When holes are present on the surface of the restoration, the pressurized air inside the restoration can be preferentially discharged through the holes, preventing issues with trapped air from affecting the adhesive filling process [34]. Moreover, the instability of AFR may be caused by variations in airflow direction, disruption of flow, and instability in the adhesive flow state caused by pressure imbalance during the bonding process.

This study found that the internal axial filling rate of all prosthesis groups stabilized at a high level of over 95% and was less affected by the diameter and position of the holes compared with the AFR at the abutment shoulder. This phenomenon can be attributed to the fact that, in practical applications, adhesive is typically applied uniformly on the inner surface of the restoration, thereby enhancing the axial filling rate. Even when the diameter of the occlusal hole is relatively large, the uniform application of adhesive can still maintain an adequate axial filling rate.

The retention strength of the prosthesis is influenced by multiple factors, including the type of adhesive, the materials used for the restoration and abutment, surface treatment, among others. Additionally, the diameter and location of the holes can influence the flow dynamics of the adhesive and the bonding area, thereby impacting the overall bonding strength. In this study, the retention strength of crowns with 2.5 mm holes was significantly reduced. This reduction may be due to the smaller bonding area of large open-hole crowns, which results in longer edge lines and increased susceptibility to damage. Larger open holes in the crown reduce the adhesive fluid pressure during bonding, leading to a non-uniform adhesive distribution and a lower shoulder AFR compared with crowns with smaller open holes.Previous studies have investigated the effect of varying hole diameters and locations on the retention strength of prostheses using temporary adhesives [19,20].In this study, we utilized a permanent resin adhesive, and the findings were more closely aligned with actual clinical application scenarios.

### Limitations

The modified dual-color silicone rubber method used in this study effectively simulates the internal AFR of restorations. However, subtle variations in rheological properties (e.g., viscosity and flow dynamics) between silicone and clinical resin adhesives still merit consideration.

## Conclusion

Different hole placement strategies could influence the AFR of implant restorations. Specifically, unopened or larger holes might decrease the AFR at the abutment shoulder, whereas the internal axial AFR of the crown was less affected by hole diameter and position. An increase in hole diameter led to a decrease in the abutment shoulder AFR and diminished the retention strength of the implant restoration.

## Supporting information

S1 Table Data of AFR(%) at abutment shoulder for each group. (XLSX)

S2 Table Data of AFR(%) of axial surface for each group. (XLSX)

S3 Table Data of Cement retention force(N) for each group in retention strength test. (XLSX)
